# Impact of multisection and immunohistochemistry in lymph node staging of Gastric Carcinoma – Case series

**DOI:** 10.1038/s41598-020-59000-8

**Published:** 2020-02-24

**Authors:** Fernando A. V. Santos, Ana P. Drummond-Lage, Alberto J. A. Wainstein, Marco A. Dias-Filho, Paulo R. Savassi-Rocha, Tulio P. Navarro

**Affiliations:** 1 Faculdade Ciencias Medicas de Minas Gerais, Alameda Ezequiel Dias 275, Belo Horizonte, MG 30130-110 Brazil; 2 Universidade Federal de Minas Gerais, Alfredo Balena 189, Belo Horizonte, 30130-100 Brazil

**Keywords:** Gastric cancer, Surgical oncology

## Abstract

Gastric carcinoma (GC) locoregional recurrence may occur even in cases where the tumor has been completely resected, possibly due to lymph node (LN) micrometastases. It is estimated that in 10% to 30% of cases, LN micrometastases are not detected by a conventional method for histological assessment of LN metastases with hematoxylin-eosin (HE). A cross-sectional study assessed 51 patients with GC by histological evaluation of the LN micrometastases through LN multi sectioning associated with immunohistochemistry analysis with monoclonal antibodies AE1 and AE3. Total gastrectomy was performed in 51% of patients. The total number of resected LN nodes was 1698, with a mean number of resected LN of 33.3 ± 13.2 per surgical specimen, of which 187 had metastasis. After the application of LN multisection and immunohistochemistry, LN micrometastases were found in 45.1% of the cases. LN staging changed in 29.4%, and tumor staging changed in 23.5% of the cases. In patients initially staged as pN0, LN staging and tumor staging changed, both in 19.2% of the cases. In patients initially staged as pN1 or more, LN staging changed in 40.0% of them, and tumor staging changed in 28.0% of the cases. The accuracy of HE for the histological staging of LN tumoral involvement was 76%, which was considered insufficient for CG patients staging. Investigation of LN micrometastasis through LN multisection and immunohistochemistry should be performed, particularly in cases where the presence of blood and lymphatic vessel invasion has been identified after conventional histological analysis, as well as in patients with advanced GC.

## Introduction

Gastric carcinoma (GC), despite the recent decrease observed in its incidence, remains the second most common cause of cancer death in the world, with more than 600,000 deaths per year. The main chance of cure of this neoplasm is on surgical resection. The standard procedure for the treatment of this condition is radical gastrectomy, which includes gastric resection with surgical margins free of neoplasia, associated with extended locoregional lymphadenectomy^1–3^.

Accurate tumor staging is one of the leading factors in the definition of the therapeutic strategy. From the exact knowledge of the extent of tumor dissemination, it is possible to define, with greater security, the best therapeutic approach for each patient, and, consequently, avoid incomplete or excessive treatments^4,5^.

Recent literature has discussed the role of lymph node (LN) micrometastasis in GC. Conceptually, micrometastases are metastases of sizes between 0.2 mm and 2.0 mm (5). Its incidence varies between 10% to 30%^6,7^. Currently, many authors admit that its presence is associated with a worse prognosis and that the clinical behavior of these patients is like those with lymph node involvement by metastasis^8^. In the submucosal GC (T1b), with the absence of LN metastases and micrometastases, the five-year survival rate is close to 100%, being significantly higher than in micrometastases positive cases (82%)^6^.

The presence of micrometastasis may be related to locoregional recurrence, as one can see in wholly resected GC initially staged without LN metastasis at traditional histological analysis using hematoxylin-eosin (HE). It is estimated that between 22% to 66% of patients with tumor stages I and II, submitted to radical surgical treatment, including individuals without LN metastasis, died due to local or distance tumor recurrence^6,9^.

The routine evaluation of LN resected during radical gastrectomy, which includes analysis of only one LN histological section with HE, can result in the non-identification of micrometastases. This may generate a suboptimal LN staging and, therefore, lead to therapeutic measures that may be insufficient for the accuracy of the tumor staging^10,11^.

Thus, to reduce the chances of GC understaging, this research has the goal to assess the presence and frequency of LN micrometastasis, analyze the accuracy between conventional histological assessment by HE and immunohistochemical (IHC) evaluation with monoclonal antibodies AE1 and AE3associated with LN multisection, and to identify subgroups at higher risk of LN micrometastases.

## Materials and Methods

### Patients

We analyzed GC patients submitted to radical gastrectomy, associated with D2 lymphadenectomy, according to the Japanese Gastric Cancer Association guidelines (JGCA)^4^. Experienced and trained oncologic surgeons performed all surgeries.

Exclusion criteria were related to patients who had previous gastric surgery and had been submitted to neoadjuvant treatment.

### Lymph node evaluation

**Figure 1:**
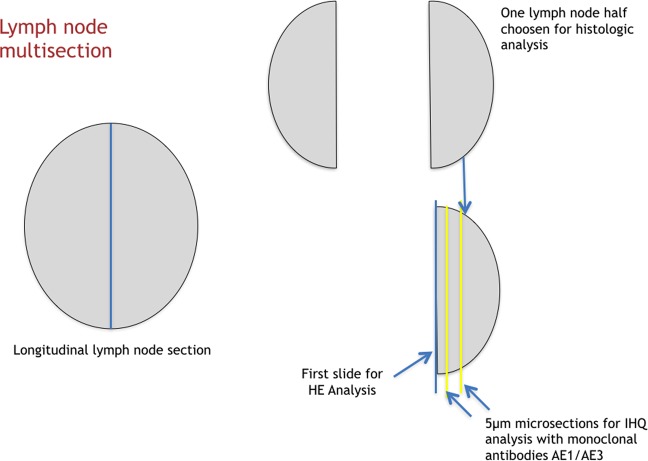
The multisection technique for histological and immunohistochemical analysis for lymph node staging.

**Figure 2:**
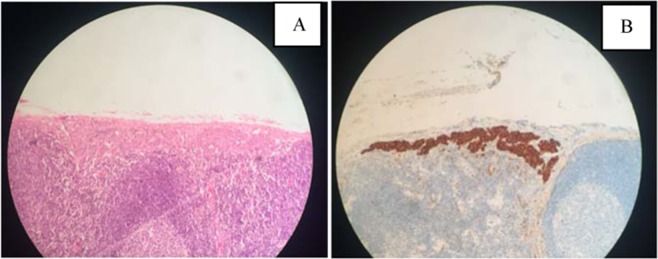
(**A**) Microscopic image of lymph node micrometastasis by Hematoxylin/Eosin. (**B**) Microscopic image of lymph node micrometastatis by Immunohistochemistry(AE1/AE3).

One pathologist examined tumor specimens. The LN involvement by metastasis and micrometastases in perigastric and extra perigastric LN nodes were analyzed. Each end every LN resected without metastases, identified by HE histological analysis, regardless of the initial LN staging, had a longitudinal cut in its structure, and one of its halves were submitted to three microsections, with an interval of 5μm among slices (Fig. 1). The first one for HE (Fig. 2A), and the other two for IHC analysis (Fig. 2B). Tissue sections from paraffin blocks were deparaffinized with xylene and rehydrated with graded ethanol dewaxed. Endogenous peroxidase was blocked with H_2_O_2_ (3%) for 30 min. Afterward, the section was incubated with primary antibody AE1/AE3 (Roche/Ventana), overnight at 4 °C. The secondary antibody used was biotinylated goat anti-polyvalent followed by streptavidin-biotin peroxidase-conjugated enzyme. The substrate and 3,3’ diaminobenzidine and section were incubated for 10 min at room temperature in the dark. The positivity of the IHQ reaction was confirmed when desmoplastic cells were identified into the LN stroma by their dark brown color.

### Definition of lymph node-positive micrometastases

Micrometastases were defined as referring to tumor cell clusters between 0.2 mm and 2.0 mm in the greatest dimension. Isolated tumors cells, which are cells or cell clusters measuring less than 0.2 mm in the greatest dimension, were not considered for statistical analysis. The GC tumor staging followed the pre-established standards from the 8th AJCC-TNM system^5^.

### Statistical analysis

For the sample calculation, considering a margin of error of 5%, 95% confidence intervals, and10% prevalence of the proportion of micrometastases in GC, a sample of 47 individuals were found. The descriptive analysis of qualitative variables was performed by absolute and relative frequencies, for quantitative variables, by the mean and standard deviation. The comparison of means between groups was performed using the Wilcoxon non-parametric test. The association between categorical variables were evaluated by Fisher’s exact test or the chi-square test, when appropriate.

## Results

The sample was composed of 51 patients, 51% females. The mean age was 62.7 ± 14.8 years. Total gastrectomy was performed in 51% of the patients. The average size of the tumors was 4.6 cm ± 2.4 cm, and predominantly in the distal location (64.7%). The total number of LN resected was 1698, with an average number of 33.3 ± 13.2 per surgical piece, of which 187 had metastasis. The average percentage of metastatic LNs was 11.0%. The tumor depth was serosa (35.3%), subserosa (11.8%), muscularis propria (21.5%), submucosa (15.7%), and mucosa (15.7%). The diffuse type, according to Laurén’s classification, was found in 41.2% of cases, followed by the intestinal type (37.2%) and the mixed type (21.6%). Blood vessels, lymphatic, and neural invasion occurred in 47.1%, 62.8%, and 35.3% of the cases, respectively.

Regarding the LN assessment by HE, in 26 cases, LN was not identified (pN0), and 25 cases had LN involvement identified on a histological assessment by HE, changing LN staging from pN1 to pN3B.

After the histological evaluation of LN with the application of IHC and LNMS, LN micrometastases were found in 23 cases (45.1%). Five cases were initially staged as pN0 by HE, and 18 cases belonged to the group of patients with known LN metastasis.

The LN staging by HE detection showed an accuracy of 76.0%, sensitivity of 78.0%, and specificity of 75.0%. In its turn, the positive and negative predictive values of histological assessment by HE in the detection of any LN metastasis were 72.0% and 80.0%, respectively.

IHC and LN MS diagnosed LN micrometastases in five cases (19.2%) out of the subgroup of 26 pN0 patients. The nodal staging changed in all these five cases (19.2%), being pN1 in four cases and pN2 in one case (Table 1).

**Table 1:** Staging and tumor restaging before and after the detection of micrometastases by IHC and MS in patients operated for gastric cancer without lymph node metastases by hematoxylin-eosin (n = 5).

Tumoral staging before MS/IHC evaluation - number of cases	Initial tumoral staging	Tumoral staging after MS/IHC evaluation - number of cases	Final tumoral staging
1	IA	0	IA
1	IB	1	IB
1	IIA	1	IIA
2	IIB	1	IIB
0	IIIA	2	IIIA

MS – Lymph node multisection, IHC – immunohistochemistry.

As to the subgroup of 25 patients with LN metastases diagnosed by HE, in 18 (72.0%) of them, micrometastases were also identified by IHC, and LN MS. Ten of these patients (40.0%) underwent a restaging of the LN involvement. In seven of them (28.0%), the tumor staging also suffered changes after evaluation with IHC and LN MS (Tables 2 and 3).

**Table 2:** Staging and restaging of lymph node involvement by micrometastases, after evaluation by IHC and MS in gastric cancer patients operated with known lymph node metastases by hematoxylin-eosin (n = 10).

Lymph node staging before MS/IHC- number of cases	Initial Lymph node staging	Lymph node staging after MS/IHQ - number of cases	Final Lymph node staging
7	pN1	0	pN1
1	pN2	7	pN2
2	pN3a	0	pN3a
0	pN3b	3	pN3b

MS – Lymph node multisection, IHC – immunohistochemistry.

**Table 3:** Tumoral staging and restaging before and after the detection of micrometastasis to IHC and MS in patients operated by gastric carcinoma with known lymph node metastasis to hematoxylin-eosin (N = 7).

Tumoral staging before MS/IHC evaluation: number of cases	Initial tumoral staging	Tumoral staging after MS/IHC evaluation: number of cases	Final tumoral staging
2	IIA	0	IIA
2	IIB	2	IIB
2	IIIA	2	IIIA
1	IIIB	1	IIIB
0	IIIC	2	IIIC

MS – Lymph node multisection, IHC – immunohistochemistry.

Among the 51 patients evaluated, LN staging has changed in 15 of them (29.4%) after LN assessment by IHC and MS. The final tumor restaging occurred in 12 cases (23.5%), i.e., in 80% of the cases in which the LN staging changed, there was also a change in the final tumoral staging (Table 4).

**Table 4:** Lymph node staging and initial and final tumor staging in the subgroup of patients reestablished after identification of lymph node micrometastases by IHC and MS techniques in gastric carcinoma (n = 15).

Patient #	Lymph node staging before MS/IHC	Tumoral staging before MS/IHC	Lymph node staging after MS/IHC	Tumoral staging after MS/IHC
2	N3a	IIIA	N3b*	IIIB**
4	N3a	IIIB	N3b*	IIIC**
11	N1	IIB	N2*	IIIA**
16	N1	IIB	N2*	IIIA**
18	N1	IIIA	N2*	IIIA
20	N1	IIIA	N2*	IIIA
25	N1	IIIA	N2*	IIIA
29	N0	IA	N1*	IB**
34	N1	IIA	N2*	IIB**
35	N0	IB	N1*	IIA**
36	N1	IIA	N2*	IIB**
39	N0	IIB	N1*	IIIA**
41	N2	IIIA	N3b*	IIIC**
45	N0	IIB	N2*	IIIA**
50	N0	IIA	N1*	IIB**

*Cases where there was a change in the category of lymph node staging, **Cases in which tumor staging was changed, MS - lymph node multisection, IHC – immunohistochemistry.

Blood and lymphatic vessel invasion at HE analysis were significantly associated with the presence of micrometastases, at an incidence of 65% (p = 0,026) and 87% (p = 0,001), respectively. Advanced gastric cancer was considered a risk factor for LN micrometastases in comparison with early gastric cancer (Table 5).

**Table 5:** Comparison of the different variables in patients submitted to radical gastrectomy about the presence of micrometastases in gastric carcinoma (n = 51).

Variables	Micrometastases		p-valor
	No n(%)	Yes n (%)	
**Gender**			0.264
Female	12 (42.9)	14(60.9)	
Male	16 (57.1)	9 (39.1)	
**Age (years)**			1.000
Mean ± standard deviation	62.8 ± 15.0	62,7 ± 14.8	
**Gastrectomy**			0.577
Subtotal	15 (53.6)	10 (43.5)	
Total	13 (46.4)	13 (56.5)	
**Tumor size (cm)**			0.239
Mean ± standard deviation	4.3 ± 2.6	5.0 ± 2.2	
**Stomach site**			0.769
Body/Proximal	9 (32.1)	9 (39.1)	
Distal	19 (67.9)	14 (60.9)	
**Serous tumor depth**			0.141
No	21 (75.0)	12 (52.2)	
Yes	7 (25.0)	11 (47.8)	
**Laurén**			0.225
Diffuse	11 (39.3)	10 (43.5)	
Intestinal	13 (46.4)	6 (26.1)	
Mixed	4 (14.3)	7 (30.4)	
**Vascular/Lymphatic/Neural invasion**			
Vascular	9 (32.1)	15 (65.2)	0.026
Lymphatic	12 (42.9)	20 (87.0)	0.001
Neural	7 (25.0)	11 (47.8)	0.141

p-test significant when <0.05.

It was shown that 60% of the advanced gastric cancer patients had micrometastases compared to 12,5% of the early gastric cancer group (p = 0,002).

## Discussion

In the Middle West, GC is usually diagnosed at an advanced stage and with LN metastases. Those metastases are an independent factor for poor prognosis, reducing patient disease-free survival and overall survival. The detection of LN metastases is critical for the correct classification of tumoral staging and should be investigated, to allow the best prognostic definition of CG, and to provide the means for the stratification of individuals who could benefit or not from adjuvant chemotherapy^1,5,12^.

The frequency of micrometastases in GC is estimated between 10% to 30%. It may vary not only by factors directly related to the tumor itself but also by the method used for their identification. It is believed that LNmicrometastases may be responsible for tumor recurrence in patients whose tumors were resected entirely, even in cases in which there were no LN metastases^13,14^.

The latest edition of the AJCC/TNM classification for GC establishes that micrometastases when found, must be notified by the acronym pNmi. However, guidelines on how and when to carry out their research were not defined, and its presence should not affect tumor and LN node GC staging^5^.

The best method for the histological diagnosis of LN micrometastases is not known, and AJCC does not provide any guidance on this subject. In the literature, there is no definition of how many LN MS must be done. Some authors propose just one cut, and others suggest the whole LN sectioning. This can impair the implementation of LN micrometastases search at pathology laboratories due to the work overload caused by this procedure^15–18^. In either way, when searching for LN micrometastases, it is recommended IHC using anti-cytokeratin antibodies as it increases the micrometastases detection rate^19,20^.

In a study published by Isosaki *et al*., the LN, resected from111 patients submitted to radical gastrectomy were analyzed for the presence of metastases and micrometastases based on HE analysis and full sectioning of the LN, resulting in the assessment of 58,430 slides The authors concluded that, when comparing the diagnostic efficacy of LN metastases based on one section, three sections, and the entire LN sectioning, the three-sectioning method was the most cost-effective of all^21^. This finding was also reported by other authors, even in tumors of different primary sites^15,22–24^.

In the present study, three LN sections were performed. It added 59 metastatic LN to the initially 187 metastatic LN found, and the incidence of metastatic LN rose from 11.0% to 14.5% (246 of 1698 lymph nodes). The global frequency of micrometastases was 45.1%. The accuracy of histological assessment using HE was 76%. The importance of LN multisection is highlighted by the fact that the micrometastases and, sometimes, the LN metastases, could only be identified in additional cuts from the LN MS. In two cases (3.9%), they were found exclusively in the third section level of the LN, and, in these cases, the LN staging changed from pN0 to pN1, and the tumor staging went from IB to IIA. Thus, if the MS had not been performed, the LN micrometastases would have never been found, and it would have been a cause of tumor downstaging.

Other studies assessing the presence of micrometastases, not only in GC but also in colonic, esophageal, head and neck, and gynecological tumors, also observed the same findings. Such studies attest that, regardless of the tumor staging, the conventional histological evaluation, which is based on just one cut in the LN structure, does not stage the metastatic LN involvement adequately^13,15,16,25^.

It is not clear for which group of patients the micrometastases research would be indicated. It is possible that the finding of specific pathological variables at the HE analysis could serve as a guide for the regular survey of micrometastases in LN resected during gastrectomy.

Considering the variables analyzed in this study (age, sex, gastrectomy type, tumor size, histological type, depth of involvement of gastric wall, tumor location, number of lymph nodes resected, lymphatic vessel invasion, blood vessel invasion, and neural invasion), only the blood and lymphatic vessel invasion showed statistical significance concerning the presence of micrometastases.

Nevertheless, when considering two different groups, one with initial and other with advanced GC, it was noted that the incidence of micrometastatic LN was significantly higher in the second group. These observations were also reported by other authors^14,22,26,27^. In the overall analysis of the results obtained in the present study, it should be noted that, with the addition of LN MS and IHC, the final LN and tumor staging changed significantly. Out of the 51 patients studied, one-third of them had their LN staging changed, and 23.5% of them had a reclassification of their final tumor staging. In 80% of the cases in which there was a change in the final LN staging, the final tumor staging changed as well.

The non-identification of LN micrometastases may interfere negatively in the analysis result of the surgical treatment of GC. It may be a cause of misunderstanding of patients’ survival rates. For instance, patients with a lower category of LN involvement could have worse survival rates compared with others in a more advanced cancer stage, probably because of the non-identification of micrometastases by HE analysis^28–30^.

Lee *et al*., in 2015, prospectively evaluated the prognostic value of micrometastases and whether these should be considered or not in the pathological LN staging of GC. Analyzing 482 patients undergoing a curative gastrectomy and examining all the resected and negative LN for metastases by HE, even in those cases in which the initial LN staging was pN1 or greater, there was a change of pathological LN staging in 15.6% of the cases. When LN micrometastases were considered in the pathological LN staging, there was a higher accuracy of results: most patients initially considered as pN2 were, in fact, pN3a. With this change, the survival curve was within the normal range and maintained throughout the years. The authors urged the need for the inclusion of micrometastases in the LN node staging system^31^.

In the present study, after the completion of the LN MS and IHC analysis, LN micrometastases were found in five of the 26 cases initially staged as pN0. The LN and final tumor staging have changed in all these cases. In four situations, the final LN staging increased to N1, and one situation increased to N2. This is extremely important and demonstrates the fragility of LN staging when using only HE.

So far, adjuvant chemotherapy is not indicated for patients whose LN micrometastases were detected. According to guidelines from the National Comprehensive Cancer Network (NCCN), as well as from the JGCA, adjuvant chemotherapy has only been proved to be beneficial in patients with advanced GC, and in patients with LN metastases, increasing both disease-free survival and overall survival^32–34^.

The study has limitations. As its primary focus regards the histological and immunohistochemical diagnosis of lymph node metastasis, there is no data related to clinical outcomes, including survival and recurrence.

## Conclusions

The application of lymph node multisection and IHC to assess lymph node staging in GC patients submitted to radical gastrectomy demonstrate that the accuracy of the conventional HE method for the histological staging of lymph node involvement is insufficient. Also, the research of micrometastases through lymph nodes multisection and IHC should be undertaken, particularly in cases in which there was the identification of the presence of blood and lymphatic vessel invasion, as well as in patients with advanced CG.

### Compliance with Ethical Standards

- All procedures performed in this study were per the ethical standards of the institutional and/or national research committee and with the 1964 Helsinki declaration and its later amendments or comparable ethical standards.
- The Universidade Federal de Minas Gerais Ethics Committee previously approved this project.
- Informed consent was obtained from all individual participants included in the study.
